# Revealing oral microbiota composition and functionality associated with heavy cigarette smoking

**DOI:** 10.1186/s12967-020-02579-3

**Published:** 2020-11-10

**Authors:** Mohammad Tahseen Al Bataineh, Nihar Ranjan Dash, Mohammed Elkhazendar, Dua’a Mohammad Hasan Alnusairat, Islam Mohammad Ismail Darwish, Mohamed Saleh Al-Hajjaj, Qutayba Hamid

**Affiliations:** 1 Clinical Sciences Department, College of Medicine, University of Sharjah, 27272 Sharjah, United Arab Emirates; 2 Sharjah Institute for Medical Research, University of Sharjah, Sharjah, United Arab Emirates; 3 University Hospital Sharjah, Sharjah, United Arab Emirates; 4 Meakins-Christie Laboratories, McGill University, Montreal, QC Canada

**Keywords:** Fagerström test, Nicotine dependence, Oral microbiota, Shotgun metagenomics

## Abstract

**Background:**

Heavy tobacco smoking, a hallmark feature of lung cancer, is drastically predominant in Middle Eastern populations. The precise links between nicotine dependence and the functional contribution of the oral microbiota remain unknown in these populations.

**Methods:**

We evaluated the composition and functional capabilities of oral microbiota with relation to cigarette smoking in 105 adults through shotgun metagenomics using buccal swabs.

**Results:**

The oral microbiota composition in our study subjects was dominated by the phyla *Firmicutes*, *Proteobacteria*, *Actinobacteria*, and *Bacteroidetes*, in addition to the genera *Prevotella* and *Veillonella,* similar to previously described westernized cohorts. Furthermore, the smoker's oral microbiota represented a significant abundance of *Veillonella dispar*, *Leptotrichia *spp. and *Prevotella pleuritidis* when compared to non-smokers. Within the smoking groups, differential relative abundance testing unveiled relative abundance of *Streptobacillus hongkongensis*, *Fusobacterium massiliense*, *Prevotella bivia* in high nicotine dependent compared to low nicotine dependent profiles based on Fagerström Test for Nicotine Dependence. Functional profiling showed marked differences between smokers and non-smokers. Smokers exhibited an enrichment of Tricarballylate utilization and Lactate racemization when compared to the non-smokers. According to their nicotine dependence, enrichment of Xanthosine utilization, p-Aminobenzoyl-Glutamate utilization, and multidrug efflux pump in *Campylobacter jejuni* biosynthesis modules were detected in the high nicotine dependent group.

**Conclusions:**

These compositional and functional differences may provide critical insight on how variations in the oral microbiota could predispose to respiratory illnesses and smoke cessation relapse in cigarette smokers. In particular, the observed enrichment of *Fusobacterium* and *Prevotella* in the oral microbiota possibly suggests an intriguing linkage to gut and lung cancers.

## Introduction

The oral microbiota is the second most complex microbial ecosystem after the gut flora, consisting of a dynamic spectrum of microorganisms residing in the oral cavity and its interaction with host genetics, diet, immune system, and many other factors [1]. The bacterial microbiome is the predominant component, with species consisting mainly of obligate aerobes such as *Neisseria* and *Rothia*, facultative aerobes such as *Streptococcus* and *Actinomyces,* and obligate anaerobes including *Firmicutes*, *Bacteroidetes,* and *Spirochaetes* [2]. The community composition, although similar amongst the buccal mucosa, gingiva, and hard palate; yet is different from the soft surfaces, saliva, and gingival plaques [3]. Saprophytic protozoa such as *Entamoeba gingivalis* and *Trichomonas tenax* and fungi such as *Candida albicans* and *Saccharomyces cerevisiae* are also native residents of oral microbiota [1].

Despite the similarities in the core microbial composition existing within oral cavities, the species may vary depending on the host's diet and nutrition, genetic predisposition, hormonal factors, antibiotic exposure, alcohol consumption, and repeated infections by pathogenic bacteria. This variation, if pathogenic, is termed dysbiosis, which can cause several alterations to the host's oral and systemic health through multiple pathophysiological processes [4, 5]. Dysbiosis has been reported to be involved in the etiology of oral diseases such as dental caries, gingivitis, and periodontitis; and systemic diseases spanning from infections to cancers, such as respiratory tract infections, gastric ulcers, irritable bowel disease, rheumatoid arthritis, infective endocarditis, and cancers [1, 4, 6].

Tobacco smoking is a well-known preventable cause of death and affects nearly every organ system of the body [7]. The oral cavity is one of the first regions exposed to cigarette smoke and is at a prime disadvantage for increased carcinogenesis, impaired mucosal immunity, and alteration of the oral microbiome [8–10]. In turn, smoking increases colonization of the oral cavity by pathogenic bacteria and reduces colonization by commensal bacteria [11, 12]. Smoking enhances biofilm formation and results in greater epithelial adherence by certain pathogens, including *Streptococcus pneumonia*, *Staphylococcus aureus*, *Streptococcus mutans*; thereby, increasing susceptibility to respiratory infections and dental caries respectively in those smokers [8, 10, 12]. Furthermore, smoking contributes to the alteration in the oxygen tension of the oral and upper gastrointestinal microenvironment that encourages persistence of microaerophilic bacteria replacing the commensal beneficial species [12, 13]. Previous studies have shown an increased prevalence of the genera *Atopobium*, *Campylobacter*, and *Prevotella* among smokers and selective depletion of certain phyla, including *Proteobacteria* [12, 14–16]. Thus, tobacco smoking creates a unique dysbiotic environment in the oral cavity, influencing the microbiota composition with far-reaching consequences in the local and systemic health of the host [8]. In this study, we intend to decipher our understanding of the oral microbiota's composition and its alteration due to tobacco smoking and smoking severity (nicotine dependence level). Further, we evaluated the metabolic capabilities of the oral microbiota using shotgun metagenomic sequencing to determine microbial biodiversity and functional capabilities that associate with tobacco smoking in the oral cavity.

## Materials and methods

### Study population

In this case–control study, we recruited participants over an eight-month period between June 2019 and February 2020 in the emirates of Dubai, Sharjah, and Ajman in the United Arab Emirates. Participants completed self-administered questionnaires that included comprehensive demographic, social, and medical history, among other lifestyle information. Tobacco smokers were defined as those individuals that reported as exclusively cigarette smokers for 5 years or more. Non-smoker controls were defined as individuals who did not report smoking cigarettes or any other tobacco products and were otherwise healthy. We excluded those who reported antibiotic or prescribed probiotic use in the past three months, and those with a preexisting respiratory illness such as asthma and chronic obstructive pulmonary disease.

We have also assessed nicotine dependence by collecting participants' self-administered Fagerström Test for Nicotine Dependence (FTND) scale, as previously described [17]. Briefly, yes or no items are scored with 0 or 1, and multiple-choice items are scored from 0 to 3. The items are summed to yield a total score of 0–10. Higher FTND scores indicate greater physical dependence on nicotine. For further validation, participants also completed the Short Nicotine Dependence Syndrome Scale [18, 19].

We conducted sample size calculation for unmatched case–control with two-sided confidence level of 95 and power of 80 with ratio of controls to cases of 1. Hypothetical proportion of cases with smoking exposure of 50 resulted in minimum of 12 sample size (cases) and a minimum of 12 sample size (controls), total sample size of 24 [20, 21]. During the data collection phase, we collected 539 buccal swabs from 428 non-smokers and 111 smokers, using Isohelix DNA/RNA Buccal Swabs (Isohelix Ltd. Harrietsham, United Kingdom) following the manufacturer's instruction (Isohelix Ltd.). Case–control matching of tobacco smokers and non-smokers group yielded 105 participants consisting of 50 non-smokers and 55 smokers. The swabs were then collected in a sterile container, stored immediately into liquid nitrogen, and then transferred to a − 80 °C freezer until further analysis. Swabs from these 105 participants were further processed for analysis. All participants in the study read and signed an informed consent, and the Research Ethics Committee at the University of Sharjah approved the study protocol.

### DNA extraction and library preparation

DNA was extracted using the Qiagen MagAttract PowerSoil DNA KF kit (Formerly MO Bio PowerSoil DNA Kit) using a KingFisher robot. DNA quality was evaluated visually via gel electrophoresis and quantified using a Qubit 3.0 fluorometer (Thermo-Fischer, Waltham, MA, USA). Libraries were prepared with the Illumina Nextera library preparation kit using an in-house protocol (Illumina, San Diego, CA, USA).

### Sequencing, data curation, and sequence processing

Paired-end sequencing (150 bp × 2) was done on a NextSeq 500 in medium-output mode. Next, shotgun metagenomic sequence reads were processed with the Sunbeam pipeline [22]. Initial quality evaluation was done using FastQC v0.11.5 (Bioinformatics Group at the Babraham Institute. Software available at: https://www.bioinformatics.babraham.ac.uk/projects/fastqc/. Processing took part in four steps: adapter removal, read trimming, low-complexity reads removal, and host-sequence read removal. First, adapter removal was done using Cutadapt v2.6 [23]. Next, trimming was done with Trimmomatic v0.36 [24] using custom parameters (LEADING:3 TRAILING:3 SLIDINGWINDOW:4:15 MINLEN:36). Then, low-complexity sequences were detected with Komplexity v0.3.6 [22]. Spurious Operational Taxonomic Units (OTUs) reads were determined and removed because they matched one of the pre-specified host/contaminant genomes or due to low complexity or quality. At the end of quality control, the median and range number of quality-filtered reads per sample was 5,062,550 and 606,459, respectively. The remaining reads were taxonomically classified using Kraken2 with the MiniKraken2_v1 database [25] and with the Genome Taxonomy Database (v. 89).

For functional profiling, high-quality (filtered) reads were aligned against the SEED database via translated homology search and annotated to Subsystems, or functional levels, 1–3 using Super-Focus [26].

### Statistical analysis

We assessed the alpha diversity with Shannon and Chao1 indices after filtering out spurious OTUs, and then the significance of diversity changes was tested with the Mann Whitney test. Next, we evaluated the beta diversity, underscoring differences across samples; a non-metric multidimensional scaling analysis was used to visualize microbiome similarities. Permutational analysis of variance (PERMANOVA) was used to test for the significance of overall microbiome differences. To assess possible compositional differences in the bacterial community, binomial models (DESEq2 R package) of the form *∼group ∼dependence* of taxonomic and subsystem level 3 features were used. P values were calculated with Likelihood Ratio Tests. False Discovery Rate (FDR) p-value adjustments using the Benjamini and Hochberg method were made to correct for multiple testing. An adjusted p-value of less than 0.05 was considered significant. All analyses were conducted in the R environment.

## Results

### Bacterial summary taxonomic composition

We analyzed buccal swab samples from 105 participants for taxonomic composition, differential abundance, and functional profiling of their oral microbiota. The subjects' characteristics in this study, such as age, gender, body mass index (BMI), ethnicity, and medical history, have been provided (Table 1).

**Table 1:** Demographics of Study Cohort

Characteristics	Smokers	Non-smokers	p-value
	(n = 55)	(n = 50)	
Age, years	30.40 (9.508, 21–62)	30.30 (11.196, 21–60)	0.961^a^
Mean (SD, range)			
Gender (M%, F%)	92.7%, 7.3%	90.0%, 10.0%	0.618^b^
Ethnicity (%)			
MENA	78.20%	76.00%	0.798^b^
Asians	20.00%	20.00%	
Africans	1.80%	4.00%	
BMI (Kg/m^2^) Mean, (IQR)	24.97, (21.22–28.91)	24.92, (21.99–27.52)	0.948^a^
Prescribed probiotics use (yes%)	0.00%	0.00%	–
Exercise (yes%)	61.10%	72.00%	0.24^b^
Animal exposure (yes%)	14.50%	20.00%	0.459^b^
Antibiotics use (past 3 months) (yes%)	0%	0.00%	–
Family history			
Cancer	12.70%	6.00%	0.241^b^
HTN	41.80%	30.00%	0.208^b^
Diabetes	50.90%	30.00%	0.03^b^
Asthma	5.50%	2.00%	0.356^b^
Household Smoker (yes%)	61.80%	30.00%	0.001^b^
Family Smoker (yes%)	65.50%	36.00%	0.003^b^
Smoking Duration Mean (SD, range)	11.80 (8.065, 5–40)		
FTND	4.82 (2.427, 1–10)		
Mean (SD, range)			
Low dependence	18.2%		
Low to moderate dependence	32.7%		
Moderate dependence	32.7%		
High dependence	16.4%		

^a^Independent t-test

^b^Chi-squared test

First, we evaluated the taxonomic composition generated from high-quality reads and classified them using the MiniKraken2_v1 database [25] as the reference database for bacteria. We aggregated taxa abundances into genera and plotted the relative abundances of the most abundant ones (Additional file 1: Fig. S1). Furthermore, we plotted the relative abundances of the most abundant taxa within the smokers' group based on their FTND score (nicotine dependence); 1−2 (low dependence), 3−4 (low to moderate dependence), 5 – 7 (moderate dependence), and ≥ 8 (high dependence) (Additional file  1: Fig. S2). Nicotine dependence was further evaluated using the Short Nicotine Dependence Syndrome Scale (NDSS-S) [18, 19]. Pearson correlation suggested a significant positive correlation between FTND and NDDS-S for smokers (r = 0.646) (p-value < 0.01) (Data not shown). Next, we estimated alpha diversity (richness and evenness) from taxonomic profiles using Shannon's diversity index and Chao1 richness estimator. No significant differences across different groups were found (Additional file 1: Fig. S3). Last, to assess the overall microbial community compositional changes, PERMANOVA was used to model the effects of smoking and nicotine dependence on oral microbiota composition. We observed a significant taxonomy difference between smoker and non-smoker groups (p-value < 0.04) and a non-significant difference based on nicotine dependence among the smoker group (p-value < 0.09).

### Bacterial differential abundance based on smoking and nicotine dependence levels

**Fig. 1:**
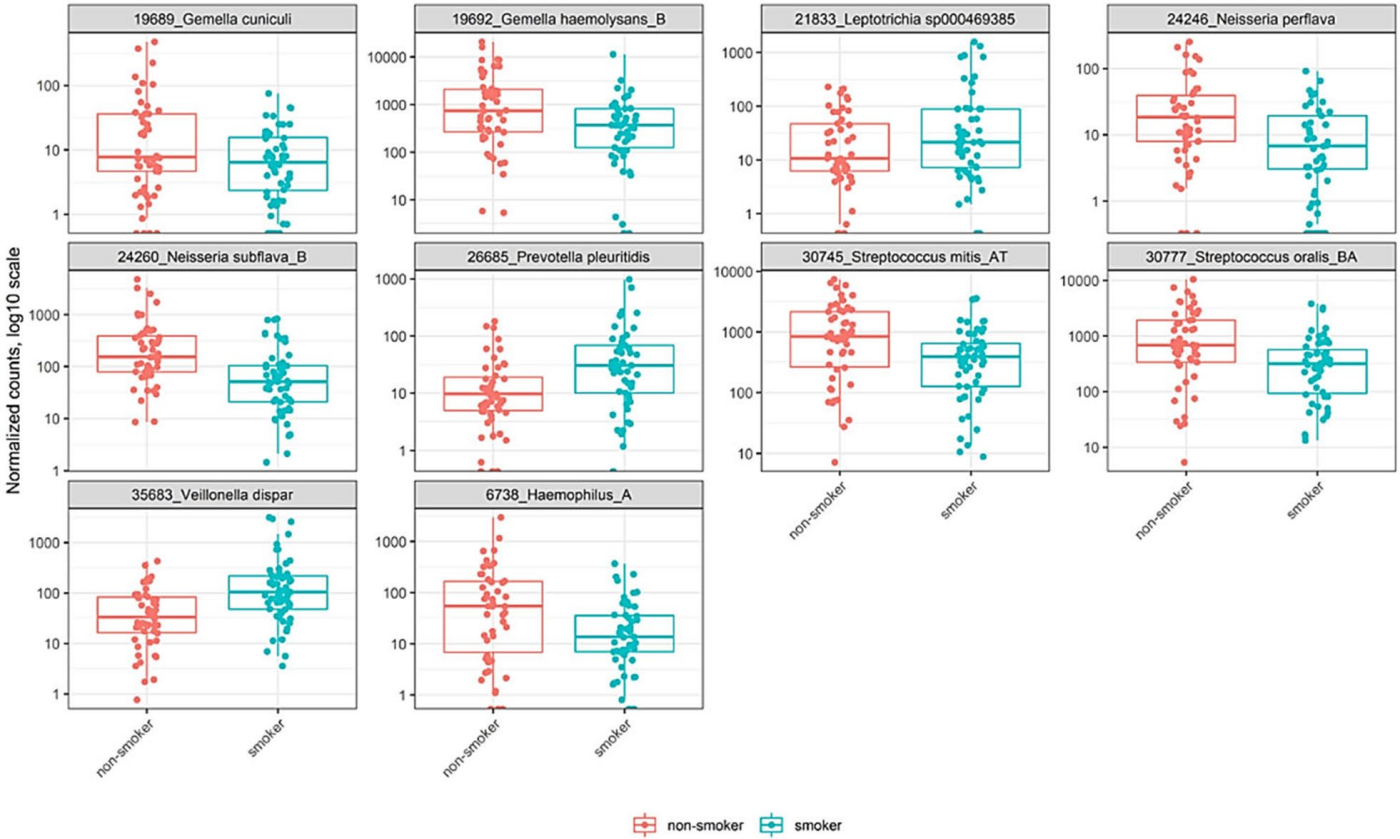
Differentially abundant taxa between smokers and non-smokers group. Panel shows relative abundance of normalized counts for the top 10 taxa. Results were calculated by negative binomial models (DESEq2 R package) of the form ∼group for differential abundance testing of taxonomic and subsystem level 3 features. P values were calculated with Likelihood Ratio Tests method. All of the above comparison are significant. Smoker and non-smoker corresponding abundance are colored in blue and red, respectively

**Fig. 2:**
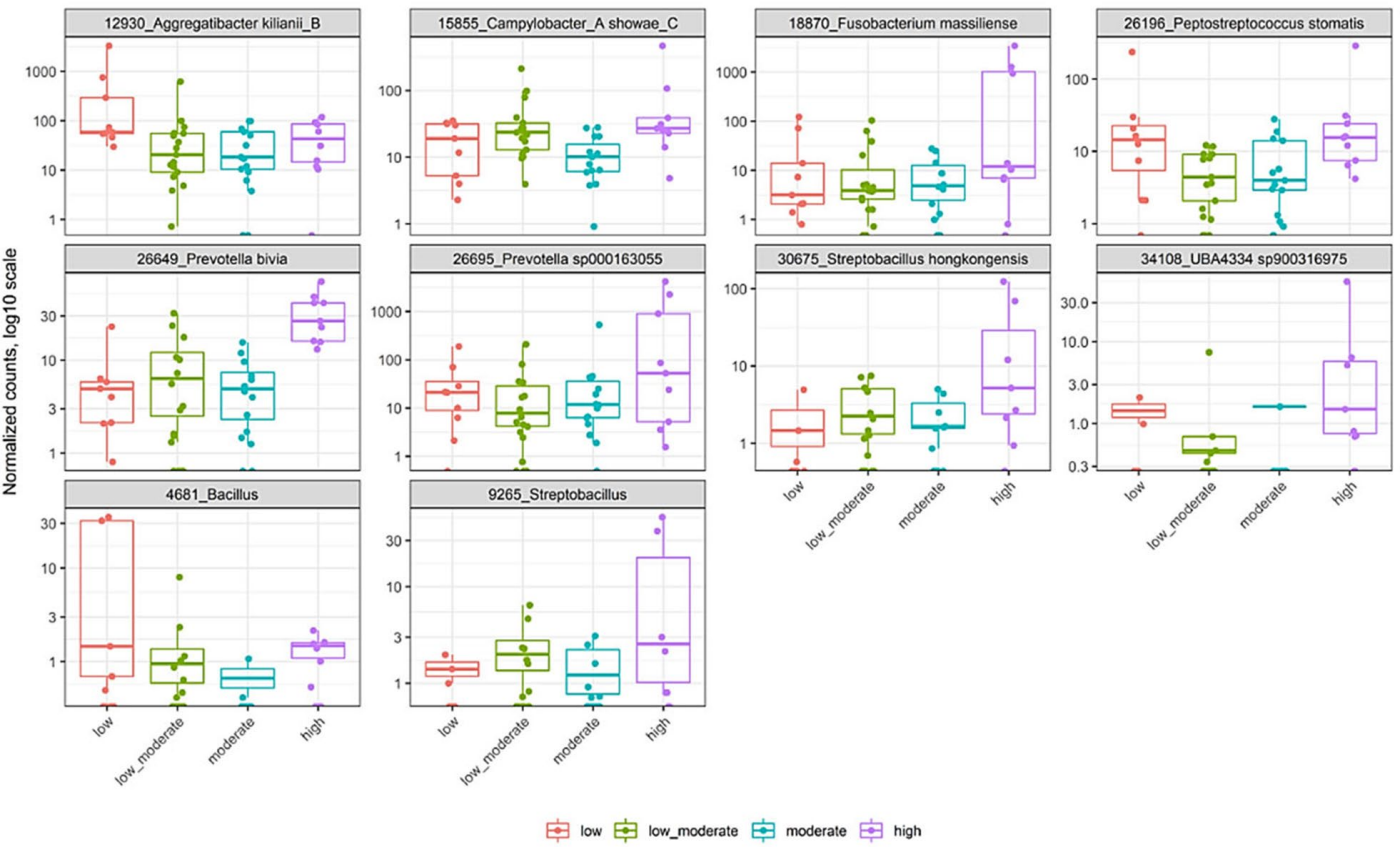
Differentially abundant taxa based on FNTD nicotine dependence score. Panel shows relative abundance of normalized counts for the top 10 taxa. Results were calculated by negative binomial models (DESEq2 R package) of the form ∼group for differential abundance testing of taxonomic and subsystem level 3 features. P values were calculated with Likelihood Ratio Tests method. All of the above comparison are significant. Nicotine dependence FTND scores; low, low to moderate, moderate, and high are colored in red, green, blue and pink, respectively

In order to further assess possible compositional differences in the bacterial community, as suggested in Additional file 1: Figure S1, we conducted negative binomial models as mentioned in methods. First, comparison of the average relative abundance between smokers and non-smokers groups revealed that profiles obtained from smokers have a statistically significant abundance of *Veillonella dispar* (Log2FoldChange 2.327, P. adjusted value < 0.0000003), *Leptotrichia* sp000469385 (Log2FoldChange 1.913, P. adjusted value < 0.0013), and *Prevotella pleuritidis* (Log2FoldChange 1.896, P. adjusted value < 0.00019). On the other hand, there was a statistically significant under-representation of *Haemophilus_A* (Log2FoldChange − 2.33, P. adjusted value < 0.00007), *Gemella cuniculi* (Log2FoldChange − 1.976, P. adjusted value < 0.00019), *Neisseria subflava_B* (Log2FoldChange − 1.87, P. adjusted value < 0.00006), *Gemella haemolysans_B* (Log2FoldChange − 1.75, P. adjusted value < 0.00085), *Neisseria perflava* (Log2FoldChange − 1.73, P. adjusted value < 0.0012), *Streptococcus oralis_BA* (Log2FoldChange − 1.56, P. adjusted value < 0.0004), and *Streptococcus mitis_AT* (Log2FoldChange − 1.39, P. adjusted value < 0.0013) in smokers (Fig. 1). We further evaluated the average relative abundance among smokers based on nicotine dependence (Fagerström score), which revealed that profiles obtained from more nicotine dependent smokers have a statistically significant abundance of *Streptobacillus hongkongensis* (Log2FoldChange 4.78, P. adjusted value < 0.00004), *Fusobacterium massiliense* (Log2FoldChange 4.63, P. adjusted value < 0.00000004), *Prevotella sp000163055* (Log2FoldChange 4.42, P. adjusted value < 0.00008), and *Prevotella bivia* (Log2FoldChange 2.46, P. adjusted value < 0.00024) (Fig. 2).

### Functional profiling of oral microbiota in smoker vs. non-smokers

**Fig. 3:**
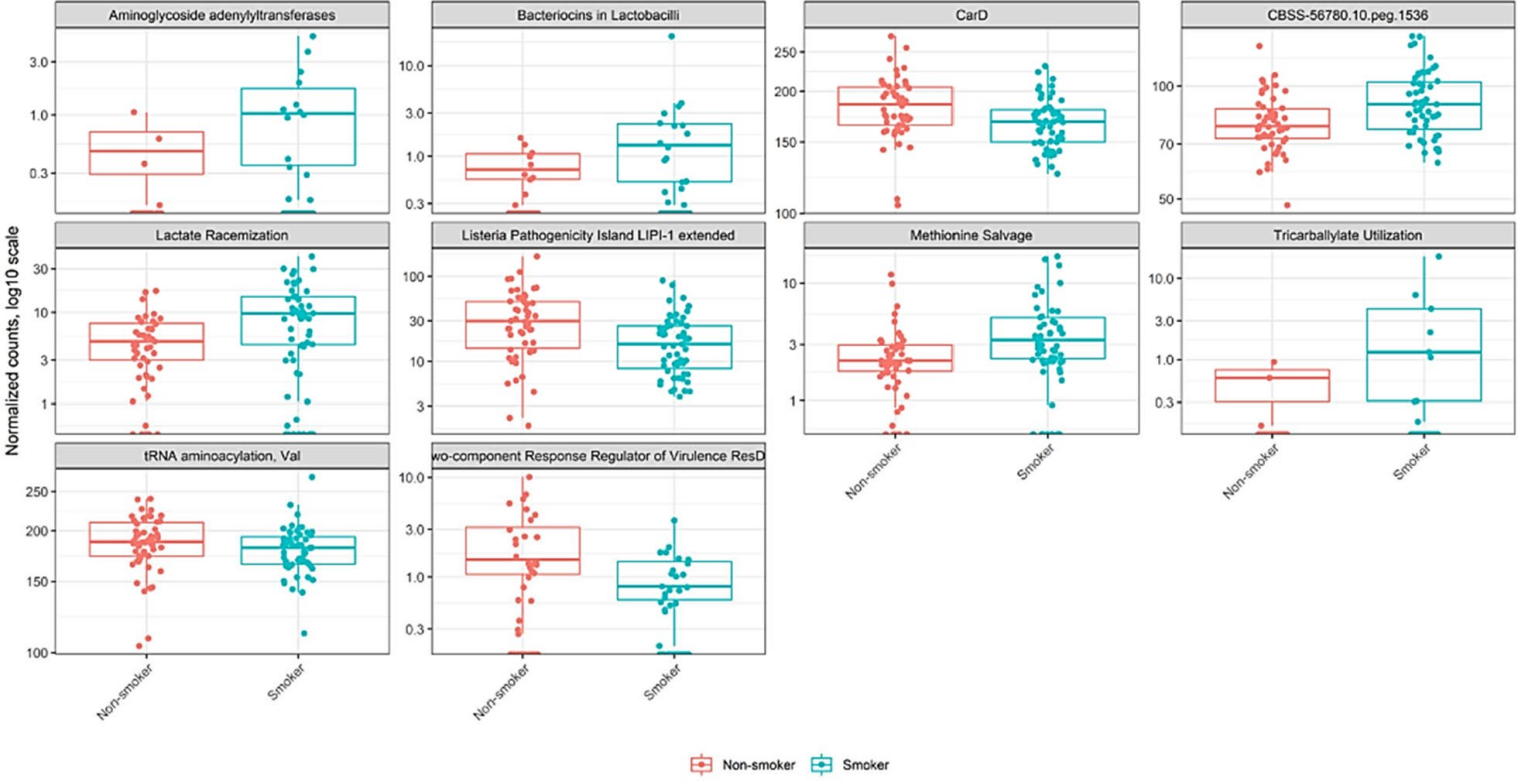
Differentially abundant gene functions of smokers vs. non-smokers group. Panel shows relative abundance of normalized counts for functional genes using SEED hierarchical categorization. All of the above comparison are significant. Smoker and non-smoker corresponding abundance are colored in blue and red, respectively

We used shotgun metagenomic sequencing to determine the functional contribution of the oral microbiota in smokers *vs*. non-smokers using the SEED hierarchical categorization. Functional profiling showed significant enrichment of Tricarballylate utilization (Log2FoldChange 2.52, P. adjusted value < 0.0013), Aminoglycoside adenylyltransferases (Log2FoldChange 2.39, P. adjusted value < 0.002), Bacteriocins in Lactobacilli (Log2FoldChange 2.29, P. adjusted value < 0.0012), Lactate racemization (Log2FoldChange 1.003, P. adjusted value < 0.0001), and Methionine salvage (Log2FoldChange 0.7, P. adjusted value < 0.0004) in smokers. It also revealed a significant depletion of Two-component Response Regulator of Virulence ResDE (Log2FoldChange − 1.28, P. adjusted value < 0.0009), Listeria Pathogenicity Island LIPI-1 extended (Log2FoldChange − 0.888, P. adjusted value < 0.00006), and CarD (Log2FoldChange − 0.139, P. adjusted value < 0.0007) in smokers (Fig. 3).

### Functional profiling of oral microbiota based on nicotine dependence severity

**Fig. 4:**
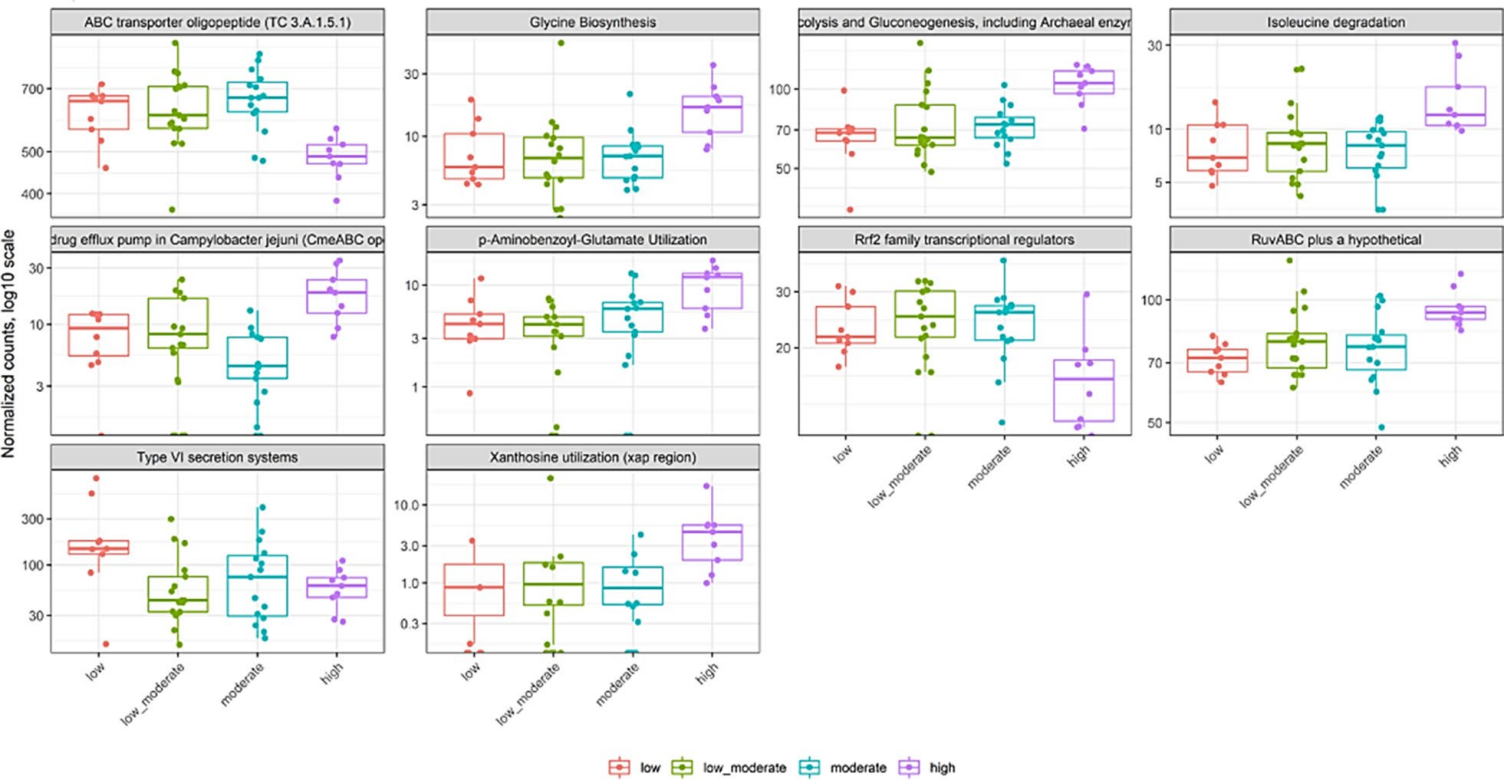
Differentially abundant gene functions based on FNTD nicotine dependence score. Panel shows relative abundance of normalized counts for functional genes using SEED hierarchical categorization. All of the above comparison are significant. Smoking dependence, low, low to moderate, moderate, and high are colored in red, green, blue and pink, respectively

Finally, we examined differentially abundant gene functions based on the Fagerström score for nicotine dependence among smokers. Pairwise functional differences determined a significant difference between low and more nicotine dependent groups (p-value < 0.02, p-value FDR < 0.05). For example, we show enrichment of Xanthosine utilization (xap region) (Log2FoldChange 3.38, P. adjusted value < 0.00007), p-Aminobenzoyl-Glutamate utilization (Log2FoldChange 1.33, P. adjusted value < 0.00056), Multidrug efflux pump in Campylobacter jejuni (CmeABC operon) (Log2FoldChange 1.14, P. adjusted value < 0.00007), Glycine biosynthesis (Log2FoldChange 1.02, P. adjusted value < 0.00062), Isoleucine degradation (Log2FoldChange 0.989, P. adjusted value < 0.00021). We also noted depletion of Type VI secretion systems (Log2FoldChange − 1.99, P. adjusted value < 0.00027), Rrf2 family transcriptional regulators (Log2FoldChange − 0.598, P. adjusted value < 0.00067), and ABC transporter oligopeptide (TC 3.A.1.5.1) (Log2FoldChange -0.351, P. adjusted value < 0.00001) in the more nicotine dependence group (Fig. 4).

## Discussion

The mouth is a highly heterogeneous ecological system with dynamic interplay between the host and oral microbiome [27]. The collective function of microbial communities is a major determinant of homeostasis or dysbiosis, and host factors such as inflammation and dietary sugars may ultimately favor health or disease such as dental caries and periodontitis [28]. In this report, we attempted to explore oral microbial profiles and functions that influence host homeostasis in the background of cigarette smoking. We explored the oral microbiota of chronic tobacco smokers in the Middle-Eastern population and described, for the first time, the functional contribution of the oral bacterial community based on nicotine dependence assessed by the Fagerström scale [17]. A final study population of 105 subjects, with an average age of 30 years, recruited in northern emirates of UAE was used for shotgun metagenomics analysis. We used buccal swabs, a more specific sampling method for the bio-adherent bacteria as compared to mouth wash sampling previously conducted in a UAE based-study [29]. Consistent with several previous reports, we detected a significant taxonomic difference between smoker and non-smoker groups, but no significant differences in terms of microbial diversity and richness, as shown in Additional file 1: Figure S3 [30–32]. Interestingly, a previous study conducted in the UAE determined only a marginal significance of the overall oral microbial differences in smokers compared with non-smokers, underscoring the geographic and ethnic contribution [29]. However, our findings were not consistent with other groups reporting a significant change in richness and diversity [33, 34]. The observed fluctuations in oral microbiota richness and diversity reporting by several groups are not unusual and further assert the high complexity and significant effects of several factors such as diet, geography, ethnicity, and host factors. That said, the oral microbiota in our study exhibit comparable dominance of phyla *Firmicutes*, *Proteobacteria*, *Actinobacteria*, *Bacteroidetes,* and genera *Prevotella* and *Veillonella* to that of oral microbiota in other populations across the globe [16, 34, 35].

Differential abundance testing of bacterial communities based on nicotine dependence scores revealed a relative abundance of *Streptobacillus hongkongensis* among more nicotine dependent smokers (high Fagerström score). Previous studies reported the isolation of *S. hongkongensis* from patients with quinsy, pneumonia, and septic arthritis [36, 37], which was later reported as part of the human oropharynx natural reservoir [38]. Increased risk of developing serious respiratory illnesses might be partly attributed to more nicotine dependent smokers. That said, we acknowledge that the overall number of reads attributed to this species is generally very low and requires further validation. Furthermore, complications of streptobacilliary infections may include endocarditis, brain abscesses, amnionitis, as well as persistent severe arthritis [39].

Smoking tobacco is the single largest risk factor for the development of lung cancers. Several studies established that *Fusobacterium nucleatum* plays a major role in colorectal carcinogenesis via Fap2 mediated binding to tumor-overexpressed Gal-GalNAc-binding lectin [40–42]. Therefore, *F. nucleatum* was deemed useful as a microbial biomarker for colorectal cancer detection [43]. Interestingly, we discovered that the phylogenetically similar *Fusobacterium massiliense,* which exhibited substantial sequence similarity with *F. nucleatum*, has a significant relative abundance among more nicotine dependent smokers. Furthermore, protein–protein BLAST analysis of the Fap2 surface protein of *F. nucleatum* ATCC 23,726 produced a significant sequence alignment with pyridoxal phosphate-dependent aminotransferase of *F. massiliense* [41, 44], the active form of vitamin B6. A previous study examined over 44,000 individuals and evaluated their smoking history and B6 vitamin supplement use over 10 years, this study found that high dosages of vitamin B6 supplements were associated with 3–4 folds increase in lung cancer risk among smokers at baseline, although the exact mechanism of this association is not yet known [45]. *Fusobacterium,* similar to *Bacteroides*, *Bifidobacterium*, *Actinobacteria*, and *Proteobacteria,* possess a vitamin B6 biosynthesis pathway. *Bacteroidetes* and *Proteobacteria* likely produce vitamin B6, starting from deoxyxylulose 5-phosphate and 4-phosphohydroxy-l-threonine [29]. Several prevailing hypotheses may explain the link. First, several B vitamins, including B6, B9 (folate), and B12 interact with homocysteine and methionine in this complex one-carbon metabolism pathway, and disruption of this process may promote carcinogenesis [46]. Second, a study reported that among B6 metabolism markers, it was the inflammation-related changes in a vitamin B6 catabolism marker, the 4-pyridoxic acid/pyridoxal plus pyridoxal 5′-phosphate ratio, which was linked to increased lung cancer risk [47]. Third, excessive supplementation of folic acid and vitamin B12 was found to be associated with changes in DNA methylation of several genes that could be reactivated or deregulated during carcinogenesis [48]. Altogether, perhaps enrichment of *F. massiliense* among more nicotine dependent smokers suggest a possible linkage to lung cancer in a pyridoxal phosphate-dependent manner. Tobacco smoking, colorectal cancer, and a high relative abundance of gut *Prevotella* were linked to each other in an intriguing association [49]. Here, we also noted an increase in the relative abundance of *Prevotella sp000163055* and *Prevotella bivia* in oral microbiota of heavy smokers, thereby suggesting a possible downstream effect on the development of colorectal cancers.

The metabolic capabilities of oral microbiota were evaluated using a shotgun metagenomic sequencing approach to determine microbial biodiversity and functional capabilities associated with tobacco smoking in the oral cavity. Functional profiling showed significant enrichment of Tricarballylate utilization among smokers *vs*. non-smokers group, a good chelator of magnesium that could lead to magnesium deficiency [50]. Magnesium plays a vital role in tobacco addiction by inhibiting several essential steps of nicotine addiction, such as dopamine secretion, NMDA receptor stimulation by glutamate, and the synthesis of substance P and nitric oxide [51, 52]. A previous study showed a significant decrease in the number of cigarettes smoked and Fagerström scores after 28 days of magnesium therapy [53]. This observation of enriched bacterial genes involved in Tricarballylate utilization among smokers suggests an intriguing role of oral dysbiosis in maintaining nicotine addiction. Moreover, a significant increase in the nickel-dependent lactate racemase enzymes was observed in smokers, consistent with the toxic nickel exposure from tobacco smoking [54, 55].

Finally, we examined the differentially abundant gene functions in correlation with the Fagerström score for nicotine dependence among smokers. Significant enrichment of xanthosine utilization was observed among more nicotine dependent smokers, which is a catabolite of purine nucleotides that leads to caffeine synthesis [56]. This enrichment could be linked to the positive association between smoking and coffee consumption, in which heavy smokers require greater coffee consumption than others to obtain an equivalent satisfactory effect of caffeine, as reported in a study of two European cohorts [57]. Lastly, we noted an enrichment of the Multidrug efflux pump in *Campylobacter jejuni* (CmeABC operon) biosynthesis module in the heavy smokers' group, an important component of bacterial virulence that can predispose heavy smokers to additional risk of tobacco-related morbidity and mortality [58]. It is important to mention that our findings need further validation on a larger cohort. The data obtained from self-administered questionnaires was subject to self-reporting bias; however, a study staff was available during the questionnaire to answer any questions.

## Conclusions

We used the shotgun metagenomics approach to shed new light on the complex functional profiles of the oral microbiota in tobacco smokers from the Middle East. To the best of our knowledge, this is the first report on oral microbiota role in heavy smoking among Middle Eastern populations based on nicotine dependence assessed by the Fagerström test. Our data identified significant compositional and functional variations in oral microbial communities, especially among the more nicotine dependent (heavy smokers) that have been linked to several respiratory illnesses and smoking cessation relapse. We hope this information may help us to understand the oral microbiome compositional changes in smokers and their impact on respiratory health and tobacco control strategies.

## Supplementary information

**Additional file 1.** **Figure S1.** Oral microbiota community composition of smokers vs. non-smoker groups. **Figure S2.** Oral microbiota composition based on Fagerström Test for Nicotine Dependence (FTND) score. **Figure S3.** Effects of smoking and nicotine dependence on oral microbiota richness and diversity.

## Data Availability

The data are all published. The data that support the findings of this study are available on request from the corresponding author.

## Abbreviations

BMI: Body mass index
FTND: Fagerström Test for Nicotine Dependence
HTN: Hypertension
MENA: Middle East and North Africa
NDDS-S: Short Nicotine Dependence Syndrome Scale
SEED: Categorization system that organizes gene functional categories into a hierarchy
SUPER-FOCUS: A Tool for Agile Functional Analysis of Shotgun Metagenomic Data
