# Supplementary material for: Revealing oral microbiota composition and functionality associated with heavy cigarette smoking
Source: J Transl Med. 2020 Nov 10;18:421. doi: 10.1186/s12967-020-02579-3 (PMC7653996; doi:10.1186/s12967-020-02579-3)
Supplement: Supplementary file 1 — Additional file 1. Figure S1. Oral microbiota community composition of smokers vs. non-smoker groups. Figure S2. Oral microbiota composition based on Fagerström Test for Nicotine Dependence (FTND) score. Figure S3. Effects of smoking and nicotine dependence on oral microbiota richness and diversity. [file 12967_2020_2579_MOESM1_ESM.pdf]

Revealing oral microbiota composition and functionality associated with heavy cigarette smoking.

Mohammad Tahseen AL Bataineh[1,2*], Nihar Ranjan Dash[1], Mohammed Elkhazendar[1], Dua'a Mohammad Hasan Alnusairat[1], Islam Mohammad Ismail Darwish[1], Mohamed Saleh Al-Hajjaj[1,3], Qutayba Hamid[2,4]

Additional file 1

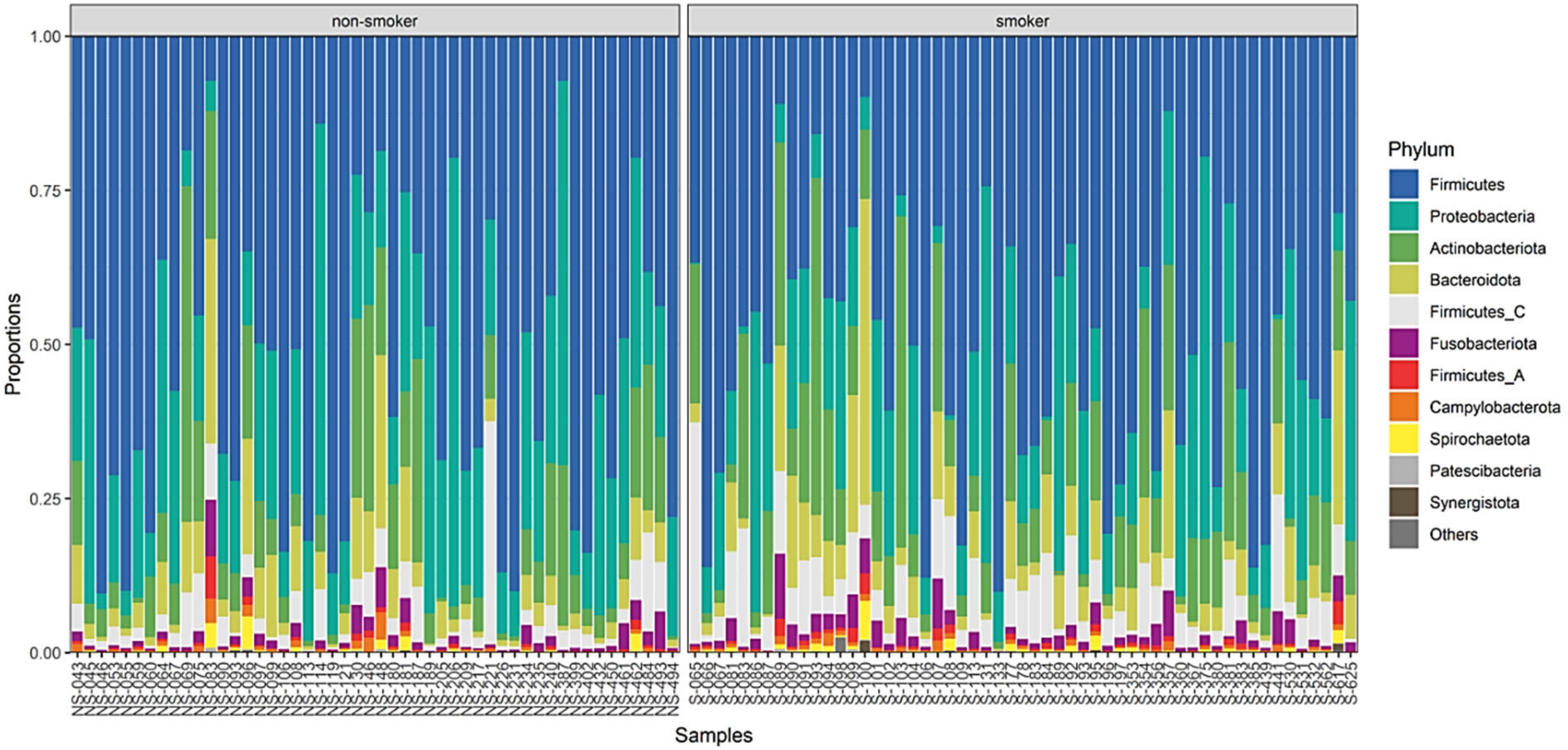

Figure S1. Oral microbiota community composition of smokers vs. non-smoker groups.

We aggregated taxa abundances into genera, and plotted the relative abundances of the most abundant ones. In the figure legends, “Other” represents lower-abundance taxa.

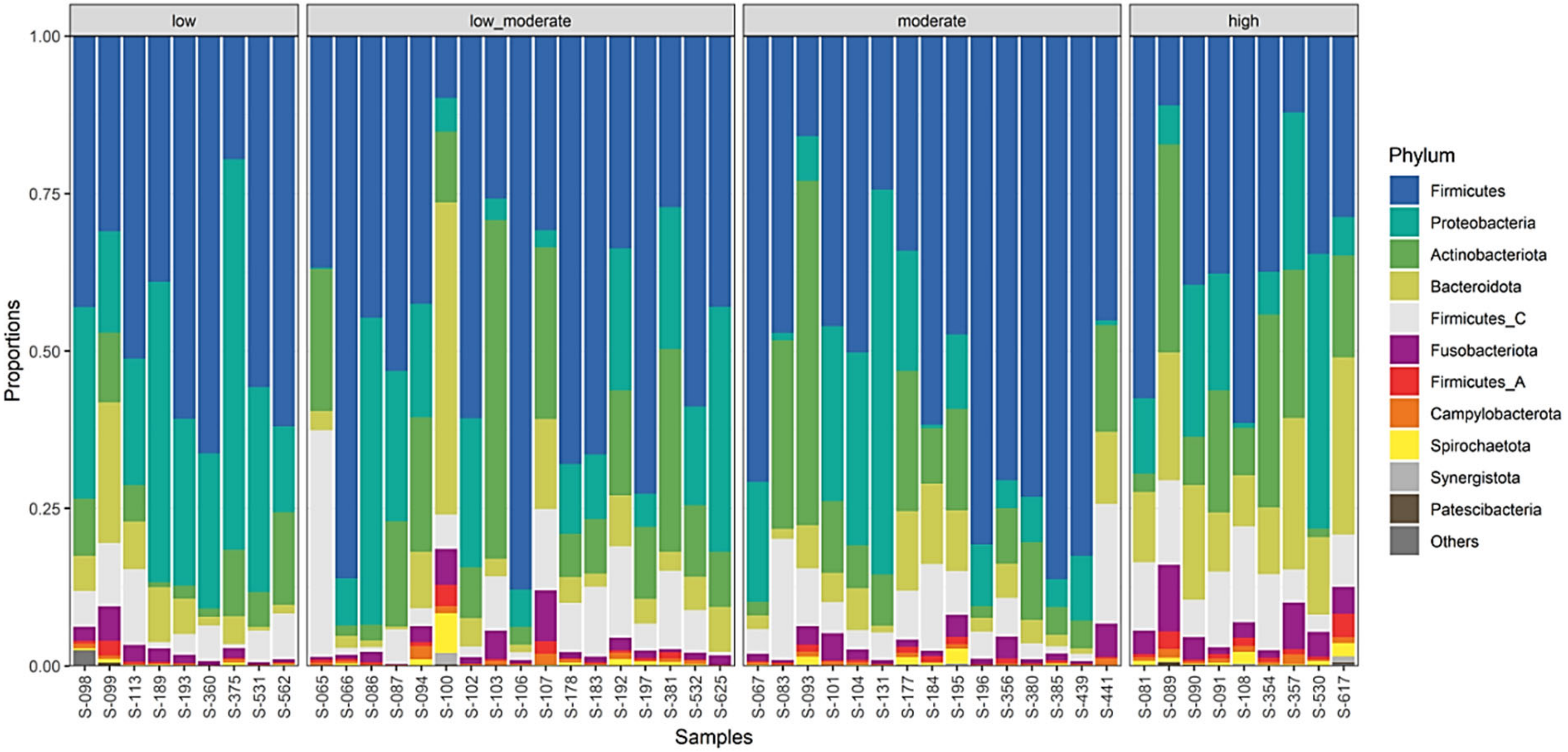

Figure S2. Oral microbiota composition based on Fagerström Test for Nicotine Dependence (FTND) score

We aggregated taxa abundances into genera, and plotted the relative abundances of the most abundant ones based on participant's FTND score; 1 − 2 (low dependence), 3 - 4 (low to mod dependence), 5 - 7 (moderate dependence), and ≥ 8 (high dependence) . In the figure legends, "Other" represents lower-abundance taxa.

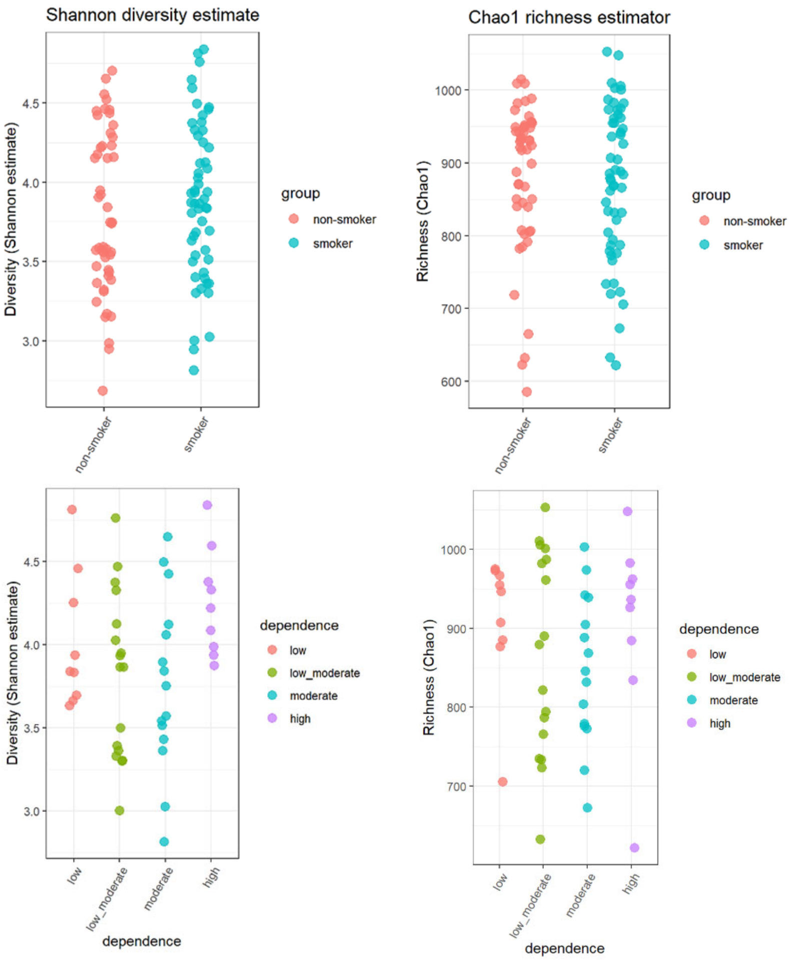

Figure S3. Effects of smoking and nicotine dependence on oral microbiota richness and diversity. Evaluation of the alpha-diversity in the 108 analyzed samples. Outlined reports the alpha diversity distributions of bacteria Shannon's diversity index and Chao1 richness estimator shows no significant differences across different groups were found.
